# Prevalence and factors associated with condom use among sexually active young women in Haiti: evidence from the 2016/17 Haiti demographic and health survey

**DOI:** 10.1186/s12905-023-02295-2

**Published:** 2023-03-29

**Authors:** David Jean Simon, Bénédique Paul, Ann Kiragu, Comfort Z. Olorunsaiye, Fanor Joseph, Ghislaine Joseph, M’Boh Delphin N’Gou

**Affiliations:** 1 Bureau d’Etudes et de Recherche en Statistiques Appliquées, Suivi et Evaluation (BERSA-SE), Port-au-Prince, Haiti; 2 Department of Agro-socio-economics, Chibas, Université Quisqueya, Port-au-Prince, Haiti; 3 Groupe d’Etude sur les Sciences de la Durabilité, Université Quisqueya, Port-au-Prince, Haiti; 4 Department of Law and Political and Social Sciences, University of Sorbonne Paris Nord, Paris, France; 5 Department of Public Health, Arcadia University, Glenside, PA USA; 6 Doctoral School of Social and Human Sciences, University of Antananarivo, Antananarivo, Madagascar; 7 Centre de Recherche Cultures Arts Sociétés (CELAT), University of Laval, Quebec, Canada; 8 Centre de Recherche de l’Institut de Démographie de l’Université de Paris (CRIDUP), Paris 1 Pantheon Sorbonne University, Paris, France

**Keywords:** Condom use, Sexually active, Young women, Risky sexual behaviors, Haiti, HDHS

## Abstract

**Background:**

Young women in Haiti remain vulnerable to sexually transmitted infections and unintended pregnancy. However, little is known about condom use among this population. This study examined the prevalence and the factors associated with condom use among sexually active young women in Haiti.

**Methods:**

Data from the 2016/17 Haiti demographic and health survey were used. The prevalence and the factors associated with condom use among sexually active young women in Haiti were assessed using descriptive statistics and binary logistic regression model.

**Results:**

The prevalence of condom use was 15.4% (95% CI 14.0–16.8). Being teenage (AOR = 1.34; 95% CI: 1.04–1.74), living in urban areas (AOR = 1.41; 95% CI = 1.04–1.90), having higher education level (AOR = 2.39; 95% CI: 1.44–4.00), being in the middle or rich category of household wealth index (AOR = 2.32; 95% CI: 1.53–3.53 and AOR = 2.93; 95% CI: 1.90–4.52), having correct knowledge of ovulatory cycle (AOR = 1.65; 95% CI: 1.30–2.10), having 2–3 lifetime sexual partners and one lifetime sexual partner (AOR = 2.04; 95% CI: 1.36–3.06 and AOR = 2.07; 95% CI: 1.35–3.17) had significantly higher odds of using condom. In addition, sexually active young women whose last partner was their boyfriend (AOR = 4.38; 95% CI: 2.82–6.81), and those whose last partner was a friend/casual acquaintance/commercial sex worker (AOR = 5.29; 95% CI: 2.18–12.85) were associated with increased likelihood of using condom compared with their counterparts whose partner was their spouse.

**Conclusion:**

The Haitian government as well as institutions involved in sexual health should consider these factors when designing sexual and reproductive health interventions targeting young women. More specifically, to increase condom use and reduce risky sexual behaviors, they should combine efforts to raise awareness and induce sexual behavioral changes at two levels. In the education system, they should reinforce sexual education in primary and secondary schools while paying special attention to rural areas. In the whole society, it is important to deepen efforts toward increased awareness on family planning and condom use, through mass media and local organizations including religious ones. Priority should be given to the poorer households, young people and women, and rural areas, in order to maximize reduction in early and unintended pregnancy, and sexually transmitted infections. Interventions should include a condom price subsidy and a campaign to destigmatize condom use which is actually a “male affair”.

## Introduction

Condom use remains the most effective means of preventing sexually transmitted infections (STIs), including the human immunodeficiency virus (HIV), and unintended pregnancy [1]. STIs are among the most common infectious diseases globally [1]. However, while they affect people of all ages, young women are disproportionally affected by adverse health outcomes [2, 3]. Recent estimates show that 374 million new cases of curable STIs occur annually, of which 75–85% occur in developing countries [4]. Furthermore, while the global unintended pregnancy rate has declined since 1990–1994 from 79 to 1000 to 64 per 1000 in 2015–2019, the proportion of unintended pregnancies ending in abortion has increased from 51 to 61% during the same periods [5]. In developing countries, particularly in sub-Saharan Africa, the burden of sexual and reproductive ill-health constitutes a huge health, social and economic burden [6]. Despite the availability of condoms, sexually active young women continue to put themselves at risk of STIs and unintended pregnancy [7]. Various studies have found a high prevalence of risky sexual behavior including unprotected sexual intercourse, sex with multiple partners, and transactional sex, particularly among university students [8, 9].

In Haiti, risky sexual behavior and its consequences are a public health concern as many young women remain at risk of contracting STIs/HIV [10]. This concern engendered an increased number of reproductive health interventions aimed at ensuring young adults have access to reproductive health information and services [11]. Although a significant decline in STIs has been observed in the last decade, two out of every seven new infections are among young people, and HIV/AIDS remains the leading cause of death among women aged 15–44 in Haiti [12]. Like all STIs, the HIV prevalence varies widely with geographic location [13]. For instance, the adult (15–49) HIV prevalence is less than 0.2% in most European countries, while it is estimated to be more than 15% in countries like Swaziland, South Africa, Lesotho, and Botswana [14]. Haiti has the highest prevalence of HIV outside of sub-Saharan Africa and leads all the other Latin American and Caribbean countries in its rates [10]. Recent estimates show that the HIV prevalence in the country is at 1.8% among adults aged 15–49, with the prevalence of infection being substantially higher for women (2.2%) than for men (1.4%) [15].

To reduce the rates of STIs/HIV infection, the international community has proposed an evidence-informed approach comprising comprehensive sexuality education [16, 17]. In addition, sexual and reproductive health and rights are included as specific targets under the sustainable development goal (SDG) 3 even though the goals are not explicit regarding the control of STIs except HIV [18]. However, despite the efforts, young women continue to have sexual intercourse without protection, exposing themselves to serious reproductive health consequences [19]. Beyond STIs/HIV and unintended pregnancy, other health consequences linked to unprotected sexual intercourse include cervical cancer as a result of human papillomavirus (HPV) infection [20], pelvic inflammatory diseases (PID), infertility, ectopic pregnancy, fetal death, and congenital infections [19]. Therefore, given these negative health outcomes on young women, and given these young women’s significant contribution to demographic dynamics, social change, and development, particular attention must be paid to them.

Studies in China have found that various factors influence condom use such as behavioral skills among college students [21] as well as environmental and structural support, perceived benefits and protection, and high safe sex self-efficacy among sex workers [22]. Studies in sub-Saharan Africa have found a strong relationship between gender inequality, restrictive gender norms, and condom use [23, 24]. In traditional societies, gender norms sustain a hierarchy of power that typically favor men creating and reinforcing their dominant position in relationship, family, and society [25, 26]. As a result, at the marital, family, and community levels, decision-making power is concentrated in the hands of men. Therefore, in sexual activity, the young woman does not have the right to refuse sex to her spouse when he wishes and the problem remains with the younger generations [27, 28]. This attitude of women stems from a culture of silence that surrounds sex pushing them to remain ignorant of sexual issues and passive in their relationships, thus leaving them with little power over the ability to negotiate their sexual protection [29]. Moreover, age at sexual debut has been associated with condom use. Evidence shows that young women who have early sexual debut are more likely to engage in risky sexual behavior [30, 31] due to a lack of confidence to negotiate condom use [32]. In the Philippines, condom use is associated with socio-demographic factors such as celibacy and greater educational attainment [33]. Similarly, in Haiti, among the few studies conducted, which did not focus on young women, it has been found that condom use increases with educational attainment of individuals [32, 34].

Still, while condom use is key to preventing the transmission of STIs/HIV and unintended pregnancy, little is known about the prevalence and factors associated with condom use among sexually active young women in Haiti. We hypothesize that demographic, social, and economic factors are more likely to influence condom use among young sexually active Haitian women. Thus this study aims to test this hypothesis and fill the gap in the literature on factors associated with condom use in Haiti.

## Materials and methods

### Data source

Secondary data from the 2016/17 Haiti Demographic and Health Survey (HDHS) were used in this study. The 2016/17 HDHS is a nationally representative survey conducted and collected as a collaboration between Haitian Institute for Children (HIC), Haiti National Bureau of Statistics (HNBS) and Haitian Ministry of Public Health (HMPH), with technical support from ICF through the DHS Program of the United States Agency for International Development (USAID). The survey was designed to provide representative estimates for main demographic and health indicators for the country as a whole, for urban and non-urban areas separately, and for each of the eleven regions in Haiti. Additional details of the HDHS methodology are outlined elsewhere [35].

### Sample design

The survey used a two-stage cluster sampling method in which at the first stage, enumeration areas (EAs) were randomly selected from the national sampling frame (i.e. the 2003 Haiti population and housing census, which was partially updated in 2011 by HNBS), and household listing. The second stage involved a systematic selection of households from the selected EAs. A total of 13,546 households were selected for the sample, of which 13,451 were occupied. Of the occupied households, 13,405 were successfully interviewed, yielding a household response rate of 99.7%. Additionally, out of 13,405 households interviewed, 14,371 women of childbearing age were successfully interviewed, with a response rate of 98.9% [35].

The 2016/17 HDHS data was divided into several sub-datasets including *Household*, *Women*, *Men*, *Births* and *Couples*. The *Women* dataset, which contains information on sexual and reproductive health of women aged 15–49, was used for our study [35]. The analytic (weighted) sample was restricted to young women (aged 15–24).

### Inclusion and exclusion criteria

All young women who declared themselves to be sexually active (i.e., those who reported having had sexual intercourse during the last 4 months preceding the survey) constituted our sample study (N = 2694). In contrast, young women who reported not having had sexual intercourse during the last 4 months preceding the survey were excluded from the study.

### Study variables

#### Outcome variable

The outcome variable was condom use, which was generated from the variable V312 (Current contraceptive method) in the *Women* dataset. DHS experts generally use this variable to estimate the prevalence of condom use among women of reproductive age [36, 37]. Condom use variable was coded “yes” if young women declared condom use during the last four months preceding the survey, and “no” otherwise.

#### Covariates

The covariates selected in this study included socio-demographic characteristics namely age (“15–19” and “20–24”), place of residence (“urban” and “rural”), region (“Ouest”, “Grand Sud”, “Grand Nord”, “Artibonite”, “Centre”, “Grand’Anse/Nippes”), religion (“Christian” and “non-Christian”), education level (“primary or less”, “secondary” and “higher”), working (“yes” and “no”), correct knowledge of ovulatory cycle (“yes” and “no”), knowledge of contraceptive method (“yes” and “no”), exposure to family planning messages (“yes” and “no”), ever been tested for HIV (“yes” and “no”), age at first sex (“less than 15”, “15–17”, and “18 or older”), relationship with most recent partner (“spouse”, “boyfriend but not living together”, and “others”), age difference between partners (“respondent is older”, “same age”, and “partner is older“), total lifetime number of sex partners (“one”, “2–3”, and “more than 3”), having children (“yes” and “no”), ideal number of children (“less than 3”, “3–4”, and “5 or more”), and frequency of alcohol consumption (“never”, “frequently”, and “hardly ever”). Socio-economic wealth index variable was a composite score measured by ownership of household items and facilities based on a DHS-generated quintile index and was categorized as poorest, poorer, middle, richer, and richest [36]. In this study, the quintile index for poorest and poorer was merged as “poor”; richest and richer was merged as “rich”; and the middle was retained as “middle”. Exposure to family planning messages was also a composite variable that was created by combining two variables: “have heard about family planning messages on radio in last few months” and “have heard about family planning messages on TV in last few months”. In the *Women* dataset, both variables were coded as “yes”, and “no”. After examining the frequency distribution of the responses, we coded it as “yes” if young women heard family planning messages through at least one of these mass media, and “no” if young women didn’t hear family planning messages through any of the mass media.

### Statistical analysis

Univariate descriptive statistics were performed to summarize socio-demographic characteristics of the study sample. Thereafter, bivariate analysis was employed to analyze the prevalence of condom use by socio-demographic characteristics. The Pearson’s chi-square statistic with its corresponding probability level was computed to assess whether there existed significant associations between the outcome and independent variables. Finally, a binary logistic regression with backward elimination selection was used to identify important factors for condom use among sexually active young women [38, 39]. The multi-collinearity was checked using the variance inflation factor (VIF) to avoid the inflation of the effect size of independent variables [40]. We found no evidence of collinearity among the explanatory variables (All VIF < 10, Mean VIF = 2.54, Min VIF = 1.03, Max VIF = 4.09) [40]. A p < .05 was considered statistically significant and adjusted odds ratios (AORs) with 95% confidence intervals (CIs) were reported.

All statistical analysis was done in STATA 14 software using *svy* command to adjust for the complex sampling structure of the data. Furthermore, data were weighted to account for the differential selection probabilities at the EAs, households, and individual levels.

### Ethics

As stated earlier, this study is based on a secondary analysis of publicly available data, under free registration and request (https://dhsprogram.com/data/available-datasets.cfm). The 2016–2017 dataset used was obtained from DHS Program with official permission as of May 3, 2022. The HDHS survey protocol obtained ethical clearance from the National Ethics Committee of Haiti and the Institutional Review Board of ICF/USAID. During the data collection, informed consent was obtained by participants and/or their legal guardians (under 16 years of age). Since this is a study involving secondary database, we were waived the need for additional informed consent. The participants’ anonymity and confidentiality were assured. All methods were carried out in accordance with relevant guidelines and regulations.

## Results

### Background characteristics of sexually active young women

Table 1 shows the socio-demographic characteristics of young women, who were sexually active in the four months prior to the survey (n = 2,694). More than two-thirds (68.3%) were aged 20–24 years and 31.7% were teenagers (15–19 years). The mean age of respondents was 20.7 years (SD ± 2.5). Most of them were christians (86.4%) and lived in urban areas (52.9%). Slightly more than 40% of them were from “Ouest” region, followed by 19.3% from “Grand Nord”, 14.3% from “Artibonite”, 13% from “Grand Sud”, 7.1% from “Grand’Anse/Nippes”, and 5.8% from “Centre”. Nearly a third (32.0%) of young women had primary education or less, 62.9% had secondary education, and 5.1% had a higher education level. Most (33.1 and 46.1%) were in the poor and rich wealth index categories respectively, 74.1% were not working, and 9.8% frequently drank alcohol.

**Table 1:** Socio-demographic characteristics of young sexually active women

Socio-demographic characteristics	All young women	
	N	Percentage
Age		
15–19 years	853	31.7
20–24 years	1841	68.3
Place of residence		
Urban	1269	47.1
Rural	1425	52.9
Region		
Ouest	1092	40.5
Grand Sud	350	13.0
Grand Nord	520	19.3
Artibonite	384	14.3
Centre	157	5.8
Grand’Anse/Nippes	191	7.1
Religion		
Christian	2328	86.4
Non-Christian	366	13.6
Education level		
Primary or less	863	32.0
Secondary	1694	62.9
Higher	137	5.1
Wealth Index		
Poor	891	33.1
Middle	561	20.8
Rich	1242	46.1
Currently working		
Yes	699	25.9
No	1995	74.1
Correct knowledge of ovulatory cycle		
Yes	732	27.2
No	1962	72.8
Knowledge of contraceptive method		
Modern method	2694	100.0
Exposure to mass media FP messages		
Yes	659	24.4
No	2035	75.6
Ever been tested for HIV		
Yes	1034	38.4
No	1660	61.6
Age at first sex		
Less than 15	586	21.8
15–17	1510	56.0
18 or more	598	22.2
Relationship with most recent partner		
Spouse	1022	37.9
Boyfriend but not living together	1634	60.7
Others	38	1.4
Age difference between partners		
Respondent is older	66	2.4
Same age	146	5.4
Partner is older	2482	92.2
Total lifetime number of sex partners		
One	984	36.5
2–3	1318	48.9
More than 3	392	14.6
Having children		
Yes	1090	40.5
No	1604	59.5
Ideal number of children		
Less than 3	1614	59.9
3–4	1025	38.0
5 or more	55	2.1
Frequency of alcohol consumption		
Never	1337	49.7
Frequently	265	9.8
Hardly ever	1092	40.5
Total	2694	100.0

All the young women interviewed declared having knowledge of modern contraceptive methods, while less than 30% (27.2%) of them had correct knowledge of the ovulatory cycle. More than three quarters (75.6%) of them had not been exposed to mass media FP messages in last few months preceding the survey, and 61.6% had never been tested for HIV. In addition, 21.8% of respondents had experienced their first sexual intercourse before age 15 and 22.2% at age 18 and above. About 4 out of 10 (36.5%) young women reported to have one sexual partner across their lifetime, and 14.6% had more than 3. Over 40% (40.5%) of respondents already had at least one child, and “less than 3 children” was the ideal number of the majority (59.9%) of them. Slightly more than a third (37.9%) of young women reported their most recent partner was their spouse, and 60.7% declared their most recent partner was their boyfriend (but not living together). Finally, 92.2% reported that their partner was older than them.

### Prevalence of condom use by socio-demographic characteristics of sexually active young women

From the table below, the overall prevalence of condom use among sexually active young women was 15.4% (95% CI 14.0–16.8). Remarkable differences were observed according to socio-demographic characteristics. The results indicated that the prevalence of condom use among teens was 20% and 13.3% among those aged 20–24 years (Table 2). The prevalence of condom use was markedly higher in urban (22.4%) than rural (9.3%) areas. This practice was most prevalent in “Grand Sud” (18.9%), “Ouest” (17.6%), “Grand Nord” (15%), and least prevalent in “Artibonite” (9.1%). As unexpected, the proportion of Christian (16.3%) youths who used condom was above those were not Christian (10.1%). Youths with primary education level or less (5.9%) and those from poor households (5.4%) recorded the lowest prevalence of condom use. About 10% (9.6%) of respondents who were working used condom, while this proportion was 17.5% among those who were not working.

**Table 2:** Prevalence of condom use by selected socio-demographic characteristics of sexually active young women

Variables	Current condom use		P-value
	Yes (N/%)	No (N/%)	
Age			***
15–19 years	171 (20.0)	682 (80.0)	
20–24 years	245 (13.3)	1596 (86.7)	
Place of residence			***
Urban	284 (22.4)	986 (77.4)	
Rural	132 (9.3)	1292 (90.7)	
Region			**
Ouest	192 (17.6)	900 (82.4)	
Grand Sud	66 (18.9)	284 (81.1)	
Grand Nord	78 (15.0)	442 (85.0)	
Artibonite	35 (9.1)	349 (90.9)	
Centre	21 (13.4)	136 (86.6)	
Grand’Anse/Nippes	24 (12.6)	167 (87.4)	
Religion			**
Christian	379 (16.3)	1949 (83.7)	
Non-Christian	37 (10.1)	329 (89.9)	
Education level			***
Primary or less	51 (5.9)	812 (94.1)	
Secondary	309 (18.2)	1385 (81.8)	
Higher	56 (40.9)	81 (59.1)	
Wealth Index			***
Poor	48 (5.4)	843 (94.6)	
Middle	85 (15.2)	476 (84.8)	
Rich	284 (22.9)	958 (77.1)	
Currently working			***
Yes	67 (9.6)	632 (90.4)	
No	349 (17.5)	1646 (82.5)	
Correct knowledge of ovulatory cycle			***
Yes	169 (23.1)	563 (76.9)	
No	247 (12.6)	1715 (87.4)	
Exposure to mass media FP messages			*ns*
Yes	111 (16.8)	548 (83.2)	
No	305 (15.0)	1730 (85.0)	
Ever been tested for HIV			*ns*
Yes	146 (14.1)	888 (85.9)	
No	270 (16.3)	1390 (83.7)	
Age at first sex			***
Less than 15	63 (10.8)	523 (89.2)	
15–17	237 (15.7)	1273 (84.3)	
18 or more	116 (19.4)	482 (80.6)	
Relationship with most recent partner			***
Spouse	28 (2.7)	994 (97.3)	
Boyfriend but not living together	380 (23.3)	1254 (76.7)	
Others	8 (21.1)	30 (78.9)	
Age difference between partners			*ns*
Respondent is older	9 (13.6)	57 (86.4)	
Same age	29 (19.9)	117 (80.1)	
Partner is older	378 (15.2)	2104 (84.8)	
Total lifetime number of sex partners			**
One	171 (17.4)	813 (82.6)	
2–3	209 (15.9)	1109 (84.1)	
More than 3	36 (9.2)	356 (90.8)	
Having children			***
Yes	47 (4.3)	1043 (95.7)	
No	369 (23.0)	1235 (77.0)	
Ideal number of children			*
Less than 3	271 (16.8)	1343 (83.2)	
3–4	138 (13.5)	887 (86.5)	
5 or more	7 (12.7)	48 (87.3)	
Frequency of alcohol consumption			***
Never	155 (11.6)	1182 (88.4)	
Frequently	48 (18.1)	217 (81.9)	
Hardly ever	213 (19.5)	879 (80.5)	
Total	416 (15.4)	2278 (84.6)	

*p < .05, **p < .01, ***p < .001, *ns n*ot significative

The results also revealed that 23.1% of youths who had correct knowledge of ovulatory cycle used condom compared to 12.6% for those who didn’t have correct knowledge of ovulatory cycle. Furthermore, condom use prevalence was 16.8% among respondents who had been exposed to mass media FP messages in last few months preceding the survey and 15% among their counterparts who had not been exposed to these messages. Similarly, the practice of condom use was slightly higher among young women who had never been tested for HIV (16.3%). With age at first sex, the highest prevalence of condom use was observed among youths who had experienced their first sexual intercourse at 18 and above (19.4%), whereas the lowest was observed among those who made their sexual debut before age 15 (10.8%). Participants whose last sexual partner was their spouse recorded the lowest proportion of condom use (2.7%) whereas those whose last sexual partner was their boyfriend (but not living together) recorded the highest (23.3%). Almost 20% (19.9%) of youths who were the same age as their partner reported used condom, while this proportion was 13.6% among those older than theirs. Similarly, youths reported to have one sexual partner across their lifetime; youths declared having no child, those whose ideal number of children was “less than 3”, and who hardly ever drank alcohol used condom the most (17.4%, 23%, 16.8%, and 19.5%, respectively). Additionally, chi-square tests showed that association of condom use and the covariates exposure to mass media FP messages, ever been tested for HIV, and age difference between partners was not statistically significant.

### Factors associated with condom use among sexually active young women

Results of the logistic regression are presented in Table 3. After adjustment, it was found that age, place of residence, education level, wealth index, having correct knowledge of ovulatory cycle, relationship with most recent partner, total lifetime number of sex partners were statistically significant at p-value < 0.05. A teenager had 1.3 higher odds of using condom (AOR = 1.34; 95% CI: 1.04–1.74) than a 20–24 years aged woman. The likelihood of using condom was 1.4 times (AOR = 1.41; 95% CI = 1.04–1.90) as high for youths from urban areas compared to those from rural areas. Young women with higher education level were 2.4 times more likely to use condom (AOR = 2.39; 95% CI: 1.44–4.00) than those who were uneducated or with primary level. Being in the middle or rich category of household wealth index was associated with increased odds (AOR = 2.32; 95% CI: 1.53–3.53 and AOR = 2.93; 95% CI: 1.90–4.52) of condom use compared to being in the poor category. Also, having correct knowledge of ovulatory cycle was associated with 65% higher odds (AOR = 1.65; 95% CI: 1.30–2.10) of condom use, whereas having children was associated with 38% lower odds (AOR = 0.62; 95% CI: 0.43–0.91) of condom use. Compared to participants with more than three lifetime sexual partners, those who had 2–3 lifetime sexual partners and one lifetime sexual partner had 2.0 (AOR = 2.04; 95% CI: 1.36–3.06) and 2.1 (AOR = 2.07; 95% CI: 1.35–3.17) greater odds of reporting condom use, respectively. Similarly, youths whose last partner was their boyfriend (but not living together) and those whose last partner was a friend/casual acquaintance/commercial sex worker were 4.4 times (AOR = 4.38; 95% CI: 2.82–6.81) and 5.3 times (AOR = 5.29; 95% CI: 2.18–12.85) more likely respectively, to use condom than those whose partner was their spouse.

**Table 3:** Logistic regression estimates for condom use by background characteristics among sexually active young women in Haiti

Variables	Coef.	Adjusted Odds Ratio (AOR)	95% CI
Age			
15–19 years	0.2944	1.34*	1.04–1.74
Ref = 20–24 years			
Place of residence			
Urban	0.3416	1.41*	1.04–1.90
Ref = Rural			
Education level			
Secondary	0.3170	1.37	0.96–1.96
Higher	0.8732	2.39***	1.44–3.99
Ref = Primary or less			
Wealth Index			
Middle	0.8423	2.32***	1.53–3.53
Rich	1.0756	2.93***	1.90–4.52
Ref = Poor			
Correct knowledge of ovulatory cycle			
Yes	0.5004	1.65***	1.30–2.10
Ref = No			
Relationship with most recent partner			
Boyfriend but not living together	1.4776	4.38***	2.82–6.81
Others	1.6658	5.29***	2.18–12.85
Ref = Spouse			
Total lifetime number of sex partners			
One	0.7271	2.07**	1.35–3.17
2–3	0.7120	2.04**	1.36–3.06
Ref = More than 3			
Having children			
Yes	-0.4730	0.62*	0.43–0.91
Ref = No			

*p < .05, **p < .01, ***p < .001, *ns n*ot significative.

## Discussion

Our study contributes to the understanding of the factors associated with condom use among sexually active young women in Haiti. To the best of the authors’ knowledge, this is the first study that documented the prevalence and population factors associated with condom use among sexually active young women in Haiti using the 2016/17 HDHS. The results from this study revealed that the prevalence of condom use among sexually active young women was 15.4% (95% CI 14.0–16.8), two times higher than the national average [35]. Also, this study has shown that condom use practice is associated with different socio-demographic factors. The findings suggested that these factors should be considered in the future policy making.

Similar to other studies [41, 42], our findings suggest that young women in union were less likely to use condom. An important explanatory factor for this observation may be gender inequality among couples. In Haiti, a patriarchal society, when women are in union, condom use is rarely under their control [43]. Considered a “male affair”, condom use during sexual intercourse is generally determined by men [44]. Additionally, many couples decrease condom use because males partners hold economic sway and refuse to use it [43]. Often, if a married woman asks her partner to use condom, she can be accused of infidelity, physically abused, or even thrown out of the house [43]. The socio-economic vulnerability of women affects their negotiation power for safer sex [45].

Results showed that adolescents were more likely to use condom than women aged 20–24. In agreement with findings from other studies [46, 47] in Africa, this observation could be due to multiple factors. With the increase in adolescent fertility rates after the earthquake that struck Haiti in 2010 [48], many UNFPA-funded family planning outreach programs primarily targeted adolescent girls [49]. Moreover, during the last decades, several teachers of human anatomy (or biology), especially those from schools in urban areas, discussed about sexuality with their students [44, 50] which could lead more young girls to negotiate condom use with their partner during sexual intercourse [32]. Another possible explanation can be the relationship of respondents with their partner. Available data indicated that 80% of teens in our sample reported that their partner was their boyfriend (but not living together), while almost half of 20–24 year olds were in union. As mentioned above, women in union in Haiti are less likely to use condom. However, it should be noted that some women in union in Haiti, opt for other modern contraceptive methods when they do not desire to have children [35].

Increased educational status of young women was associated with condom use, which is supported by past study in Sierra Leone [51]. Education is correlated with better awareness and access to health information and resources, including available reproductive health services [51, 52]. Thus, schooling may ultimately lead to improved sexual health behavior among young people in some instances. Moreover, education has been shown to empower women through career opportunities that often translate into higher socioeconomic status, which, in turn, may increase women’s autonomy and ability to negotiate safer sex practices, including condom use [32].

Wealth index is also a strong predictor of condom use. Female youth from rich households had significantly higher odds of using condom as compared to those from poor households. Earlier studies have reported similar findings [47, 53, 54]. One possible reason is that youth from rich households have more access to modern contraceptives than those from poor households because they could afford both the direct and indirect costs of condom use [55]. Furthermore, youth from rich households are more educated [36]; therefore they are also more likely to understand information relating to condom uptake [47]. Another possible reason might be that female youth from poor households were more likely to engage in risky sexual behavior compared to those from rich households [53, 56].

Consistent with previous studies in South Africa [53], in Malawi [57] and in China [54], female youth from urban areas were more likely to use condom. Regional disparities explain this difference. Rural youth mostly come from very poor households residing in areas where roads are difficult to access, and have generally lower education levels [35, 58, 59]. In such a setting, they may experience more difficulty, for a variety of financial, social and cultural reasons, in accessing family planning services than their urban counterparts [51, 53]. Furthermore, in some rural areas, condoms can be freely obtained from family planning clinics and hospitals [60]. Given that young women in rural areas may hold more conservative attitudes toward sexual behaviors than their urban counterparts, they may feel more uncomfortable procuring condoms at these facilities for fear of being judged [43].

Having correct knowledge of ovulatory cycle was associated with greater odds of condom use. The positive effects of correct knowledge of ovulatory cycle on condom use is supported by other studies [61, 62]. The likelihood of using condom to prevent unintended pregnancy during the ovulatory phase may be higher among women who had correct knowledge of their ovulatory cycle than among those who didn’t have correct knowledge [63].

In contrast, having children was associated with reduced condom use. This finding is in line with previous studies [64, 65] but contradicts the findings of these studies [55, 66]. This could be due to the fact that, the majority of participants who reported having children were 20–24 years, came from rural areas, had primary education or less, and were in union. As discussed above, youths with these socio-demographic profiles were less likely to use condom.

Finally, our study found that young women with more than three lifetime sexual partners were less likely to use condom. In line with a study in Malawi [67], this result could be partly attributed to the fact that young Haitian women who have already had several sexual partners (in a context of generalized poverty) could be more exposed to the risks of STIs, including HIV [43]. In order to prevent STIs, they would be more likely to use condom [68].

## Limitations

There are some limitations to this study. The validity of self-reports on condom use is always a concern among young women because the respondents may be reluctant to report accurately their sexual behaviors due to social desirability bias. Hence, the results may not reflect reality. Secondly, the study was limited by the use of secondary data restricting study variables. Thirdly, this cross-sectional study could not infer causality between different predictors and condom use. Lastly, this study only focused on young women. However, it is crucial to research factors that influence condom use among males, given the male dominance in sexual relationships.

## Conclusion

The prevalence of condom use among young sexually active women in Haiti is very low and varied remarkably by socio-demographic factors. Factors associated with condom use among these young women included age, place of residence, education level, wealth index, having correct knowledge of the ovulatory cycle, relationship with most recent partner and total lifetime number of sex partners. Hence, reinforcing comprehensive sexuality education in schools, particularly information on condom practices is crucial in Haiti. Besides condom promotion programs, there is a need to continue advocating for their effectiveness and appropriate adjustments may have to be made to increase access to condoms. Specifically, our results suggest that to increase condom use and reduce risky sexual behaviors, the government and its public health partners should combine efforts to raise awareness and induce behavioral changes at two levels. In the education system, they should reinforce sexual education in primary and secondary schools while paying special attention to rural areas. In the whole society, it is important to deepen efforts toward increased awareness on family planning and condom use, through mass media and local organizations including religious ones. Above all, they should prioritize the poorer households, young people and women, particularly those living in rural areas in order to maximize reduction in early and unintended pregnancy, and sexually transmitted infections. While focusing on rural areas and poor households, interventions should include a condom price subsidy and a campaign to destigmatize condom use which is actually a “male affair”. Finally, qualitative studies are required to better understand socio-cultural barriers that impinge on the use of condoms among this population in Haiti.

## Data Availability

The dataset used in this study is publicly available, under free registration and request, on the following repository: https://dhsprogram.com/data/dataset/Haiti_Standard-DHS_2016.cfm?flag=0.
